# Biocompatibility of graphene oxide nanosheets functionalized with various amino acids towards mesenchymal stem cells

**DOI:** 10.1016/j.heliyon.2023.e19153

**Published:** 2023-08-19

**Authors:** Ali Mohammad Tamaddon, Rahman Bashiri, Haniyeh Najafi, Khadijeh Mousavi, Mahboobeh Jafari, Sedigheh Borandeh, Mahdokht H. Aghdaie, Mina Shafiee, Samira Sadat Abolmaali, Negar Azarpira

**Affiliations:** a Pharmaceutical Nanotechnology Department, Shiraz University of Medical Sciences, Shiraz, PO Box 71345-1583, Iran; b Center for Nanotechnology in Drug Delivery, Shiraz University of Medical Sciences, Shiraz, PO Box 71345-1583, Iran; c Food and Drug Administration, Isfahan University of Medical Sciences, Isfahan, Iran; d Transplant Research Center, Shiraz University of Medical Sciences, Mohammad Rasoul-Allah Research Tower, Shiraz, PO Box 7193711351, Iran

**Keywords:** Graphene oxide, Cytotoxicity, Genotoxicity, Amino acid, Mesenchymal stem cell

## Abstract

Graphene and its derivatives have gained popularity due to their numerous applications in various fields, such as biomedicine. Recent reports have revealed the severe toxic effects of these nanomaterials on cells and organs. In general, the chemical composition and surface chemistry of nanomaterials affect their biocompatibility. Therefore, the purpose of the present study was to evaluate the cytotoxicity and genotoxicity of graphene oxide (GO) synthesized by Hummer's method and functionalized by different amino acids such as lysine, methionine, aspartate, and tyrosine. The obtained nanosheets were identified by FT-IR, EDX, RAMAN, FE-SEM, and DLS techniques. In addition, trypan blue and Alamar blue methods were used to assess the cytotoxicity of mesenchymal stem cells extracted from human embryonic umbilical cord Wharton jelly (WJ-MSCs). The annexin V staining procedure was used to determine apoptotic and necrotic death. In addition, COMET and karyotyping techniques were used to assess the extent of DNA and chromosome damage. The results of the cytotoxicity assay showed that amino acid modifications significantly reduced the concentration-dependent cytotoxicity of GO to varying degrees. The GO modified with aspartic acid had the lowest cytotoxicity. There was no evidence of chromosomal damage in the karyotyping method, but in the comet assay, the samples modified with tyrosine and lysine showed the greatest DNA damage and rate of apoptosis. Overall, the aspartic acid-modified GO caused the least cellular and genetic damage to WJ-MSCs, implying its superior biomedical applications such as cell therapy and tissue engineering over GO.

## 1 Introduction

Carbon-based nanomaterials are one of the most attractive nanotechnology-based structures due to their wide range of applications [1]. Among these materials, graphene, a two-dimensional (2-D) carbon nanostructure, has received a lot of attention due to its unique physicochemical features and potential biomedical applications such as drug delivery, bioimaging, regenerative medicine, and anti-cancer therapy [2]. In contrast to graphene with limited dispersibility in biological media, graphene oxide (GO) consisting of chemically exfoliated graphene sheets is greatly dispersible due to the presence of oxygenated functional groups such as epoxide, carboxyl, and hydroxyl groups. This has encouraged researchers to utilize GO for biological applications including detection, diagnostics, imaging, and drug delivery. GO has several noticeable features such as high loading ability, unique shape and geometry, low cost, and simple synthesis, which has resulted in its widespread use in pharmaceutical and medical fields in recent years [3]. However, the rapid development of products containing GO has raised concerns about the risk of human and environmental exposure. Therefore, their safety assessment became critical, and several investigations thoroughly examined the toxicity potential of GO in detail [2]. For example, Kryuchkova et al. identified GO as a toxic material and showed that kaolin nano clay effectively mitigated GO toxicity. They reported that kaolin coagulates with GO in water resulting in a relatively large complex that minimizes the GO adverse effects [4]. Pieper et al. investigated the GO cytotoxicity in HeLa cells and demonstrated that surface-bound endoperoxide groups are responsible for GO-induced oxidative stress. Also, they suggested that UV light irradiation could reduce GO cytotoxicity [5]. Patlolla et al. claimed that carbon nanotubes (CNTs) significantly increase reactive oxygen species production and DNA damage [6]. Furthermore, Jia et al. reported that GO had higher cytotoxicity than graphene in terms of DNA damage and ROS production [7].

GO concentration and physicochemical properties such as size, shape, layer number, aggregation, purity, and surface chemistry play an important role in cytotoxicity, genotoxicity, apoptosis, autophagy, and immune responses induced in treated cells [3,8]. According to Seabra et al., the most prominent cytotoxicity mechanism of graphene is ROS production in target cells [9]. Also, Gies et al. investigated the effect of various processing procedures on GO morphology and cytotoxicity and found that GO morphology has a substantial influence on cytotoxicity in U-87 MG and HepG2 cells [10]. According to Akhavan et al., GO nano-ribbons can infiltrate cells and cause DNA fragmentation as well as chromosomal aberrations even at low concentrations and after a short exposure time [11]. Because of its wholly carbon and aromatic network, the open surface of GO can be easily modified with biomolecules such as multifunctional natural metabolites or amino acids through covalent or non-covalent interactions [12]. Amino acids are cheap and environmentally friendly, making them appropriate nucleophilic reagents. Nucleophilic and condensation reactions occur between the amine groups of amino acids with epoxy and carboxylic acid groups on the GO's surface [12]. In addition, mesenchymal stem cells (MSCs) have received much attention for their broad spectrum of rapid growth, differentiation, and clinical potential in the fast-growing field of cell therapy, tissue engineering, and production of a plethora of useful growth factors and cytokines [13]. Researchers have recently used GO to enhance the proliferation and differentiation potential of MSCs. It has been shown that the oxygen-containing functional groups of GO can influence extracellular matrix (ECM) protein adsorption, thereby promoting cell adhesion and proliferation [14,15]. Wei et al. showed that 0.1 μg/ml GO could significantly promote the proliferation of bone marrow-derived mesenchymal stem cells (BMSCs). However, increasing the GO concentration in range of 1 and 10 μg/ml could inhibit cell proliferation [16]. In another study, Lee et al. compared the effects of graphene and GO on promoting MSC differentiation towards osteoblast and adipocytes. Their findings suggested that both graphene and GO could induce osteogenic differentiation. In addition, graphene inhibited adipogenesis whereas GO strongly promoted adipogenesis due to high GO affinity for insulin, the key inducer of adipogenesis [17].

Lee et al. reported that changing the diverse surface properties of graphene nanomaterials can easily impact the unique behaviors of stem cells [18]. The current study aimed to evaluate the cytotoxicity and genotoxicity of graphene oxide (GO) freshly synthesized and functionalized with various amino acids such as lysine (Lys), methionine (Met), aspartate (Asp), and tyrosine (Tyr). Trypan blue, Alamar blue and Annexin-PI tests were used to examine the cytotoxicity and apoptotic induction of bio-functionalized GO in human fetal umbilical cord MSCs. In addition, the comet (single-cell gel electrophoresis) and karyotyping techniques were used to look into potential DNA and chromosomal damage.

## 2 Materials and method

### 2.1 Chemicals

Graphite powder (particle size = 70 μm, purity = 99.99), ethanol, EDTA (ethylene diamine tetra acetic acid), NaCl, NaOH, NaH_2_PO_4_, Tris-HCl, and Triton X-100 were purchased from Merck (Darmstadt, Germany). l-lysine (Lys), l-Methionine (Met), l-Tyrosine (Tyr), l-Aspartic acid (Asp), KMnO_4_, H_2_SO_4_, 37% hydrochloric acid (HCl), NaNO_3_, 30% hydrogen peroxide (H_2_O_2_), NaOH pellet, low melting agarose (LMA), Na_2_HPO_4_, KCl, ethidium bromide (EtBr), and Trypan blue were supplied by Sigma-Aldrich (St Louis, MO, USA). Normal melting agarose [19] and FITC-labeled Annexin V were supplied by Mahboub Bioresearch (Iran) and Cinnagen Co. (Iran), respectively. Alamar blue viability assay kit was purchased from G-biosciences (St Louis, MO, USA). All aqueous solutions were freshly prepared with deionized water (Direct Q UV3, Millipore, USA).

### 2.2 Synthesis and functionalization of graphene oxide (GO)

GO was prepared using Hummer's technique, as previously reported [20]. Briefly, 500 mg Graphite powder was added to an ice-cooled mixture of 12 ml concentrated H_2_SO_4_ and 250 mg NaNO_3_. The solution was then slowly supplemented with 1.5 g KMnO_4_ while the mixture temperature remained below 20 °C. Following complete addition, the temperature of the final solution was raised to 35 °C while mixing for 30 min. The reaction mixture was then added to distilled water (25 ml), and the temperature was increased to 98 °C and maintained for 45 min. The reaction was finally diluted with water (70 ml), followed by the addition of 2 ml of H_2_O_2_ (30%). Subsequently, the reaction color changed to bright yellow. The mixture was then centrifuged (4000 rpm, 10 min) and washed with 10% HCl along with distilled water. The resulting GO was sonicated (1 h) and dried at 60 °C to obtain pure GO sheets. In the next step, **v**arious amino acids (Lys, Tyr, Met, and Asp) were attached to GO using the previously described method [21]. In a round flask, 100 mg of GO powder was dispersed in 10 ml of deionized water. This GO suspension was treated with a solution of various amino acids (300 mg) and an equimolar quantity of NaOH in 10 ml distilled water. After 48 h of stirring at room temperature, the mixture was centrifuged, washed several times with H_2_O/ethanol, and lastly freeze-dried.

### 2.3 Characterization of functionalized GO

2.3

The Fourier transform infrared (FT-IR) analysis of various GO-amino acids vs. unmodified GO and graphite was carried out using an infrared Vertex 70 (Bruker, Germany) at 400 –4000 cm^−1^. The Raman spectra functionalized GO were recorded using a Nd:YLF laser source at λ꞊532 nm on an Almega Thermo Nicolet Dispersive Raman Spectrometer from 500 to 3500 cm^−1^. Elemental analysis (CHNS) was performed using ECS 4010 CHNS–O elemental analyzer. FE-SEM (field emission scanning electron microscopy) was utilized to observe the morphology of different amino acid functionalized GO nanosheets (HITACHI S-4160, Japan). Hydrodynamic diameter and zeta potential measurements of various amino acid functionalized GO and unmodified GO nanosheets were carried out by DLS (dynamic light scattering) method employing the Zetasizer 3000HSA (Malvern, UK).

## 3 Cellular assays

### 3.1 Cell isolation and culture

Cellular experiments were carried out on WJ-MSC (passage numbers of 3–5) isolated from fresh human umbilical cord (UCs). The cells were cultured in DMEM-F12 medium supplemented with 10% FBS (PAA, Austria) and 1% antibiotic-antimycotic solution (Penicillin-Streptomycin, PAA, Austria) at 37 °C with 5% CO_2_.

Wharton's jelly-MSCs were isolated and expanded according to the previously reported methods [22,23]. Briefly, fresh human umbilical cords (n = 3) were collected from the Obstetric Department affiliated with Shiraz University of Medical Sciences (SUMs), Shiraz, Iran. Informed consent was obtained from mothers, and the study was approved by the institutional ethics committee at SUMS, Shiraz, Iran. Hepatitis C and B virus (HCV, HBV) and human immunodeficiency virus [24] testing were used to rule out infectious cases. The tissues were stored in PBS solution supplemented with 1% antibiotic–antimycotic solution on ice. Wharton's jelly was scraped from the amnion and divided into 2–3 mm pieces after blood vessels were removed. These tissue explants were grown at 37 °C with 5% CO_2_ in DMEM-F12 supplemented with 10% FBS and 1% antibiotic-antimycotic solution. To determine the phenotype of cell-surface antigens, cells from the third passage were labeled with FITC conjugated antibodies specific for hematopoietic lineage markers CD34 and CD45, and stromal surface markers CD90 and CD44. The labeled cells were resuspended in PBS before being analyzed with a FACSCalibur flow cytometer (Becton Dickinson, USA). At least 10,000 events were recorded for each sample as similarly reported elsewhere [25].

### 3.2 Cell morphology and cytotoxicity assays

Cell morphology of GO-amino acids were assessed first by optical light microscope. WJ-MSCs were seeded (1 × 10^4^ cells/well) in 12-well plates. After 24 h incubation at 37 °C and 5% CO_2_, the cells were treated with unmodified GO or GO-amino acids at the concentration of 100 μg/ml for 48 h. To perform Trypan blue exclusion assay, WJ-MSCs were seeded (2 × 10^4^ cells/well) in 6-well plates (n = 3). After 24 h incubation at 37 °C and 5% CO_2_, the cells were treated with unmodified GO and GO-amino acids at the concentrations of 1, 2, 5, 10, 20, 50, and 100 μg/ml for 48 h. Next, the cells were trypsinized and suspended in PBS containing Trypan blue and counted by a hemocytometer to determine the percentage of viable and dead cells. The ratio of non-viable cells to total cells was used to calculate cytotoxicity that was compared to control (untreated cells) [26]. The viability of WJ-MSCs treated with various concentrations (1, 2, 5, 10, 20, 50, and 100 μg/ml) of unmodified GO and GO-amino acids was also determined using Alamar blue assay [27]. After 48 h incubation, the culture medium was aspirated, the wells were washed with sterile PBS buffer (pH = 7.4) and incubated with 10% (v/v) fresh Alamar blue for further 2 h. The fluorescence intensity was read by the fluorescence plate reader (Infinite 200, Tecan, Austria) at the respective excitation and emission wavelengths of *λ*_max_ = 545 and 590 nm.

### 3.3 Apoptosis and necrosis measurements

WJ-MSCs were seeded (2 × 10^4^ cells/well) in 6-well plates and treated with unmodified GO and GO-amino acids at the corresponding concentration of 100 μg/ml for 24 h (n = 3). Cells were harvested and washed with PBS and apoptosis/necrosis was determined by flow cytometry (BD FACS Calibur, USA) after double-staining with FITC-labeled Annexin V/PI.

### 3.4 Alkaline comet assay

The comet assay was carried out to detect DNA fragmentation as a result of its mobility in an electrophoretic field according to a previously published method [28]. First, suspensions of mesenchymal stem cells (1 × 10^6^ cells/ml) were mixed with 1% LMA at 37 °C and then placed on the slides pre-coated with 1% NMA and the slides were placed flat for 5 min in the dark at 4 °C. The prepared slides (n = 3) were placed in a cold lysis solution containing 2.5 M NaCl, 1% Triton X-100, 10 mM Tris, and 100 mM EDTA (pH = 10) for 40 min in dark, then washed with distilled water to remove the lysis solution. The slides were placed in the electrophoretic buffer containing 1 mM EDTA and 300 mM NaOH (pH = 13) for 30 min. After the electrophoresis (20 V for 40 min), the alkali in the gels was neutralized by rinsing the slides in 0.4 M Tris-HCl (pH = 7.5) for 5 min. The slides were air-dried at room temperature. All slides were stained with EtBr staining solution (20 μg/ml). The image of one hundred randomly selected cells of each slide was taken at 400 × magnification using a fluorescent microscope (Eclipse E600, Nikon). The comet scoring was performed based on the percentage of the DNA in the tail and tail moment by the ImageJ plugin OpenComet.

### 3.5 Karyotyping

For evaluation of genotoxicity of unmodified GO and GO-amino acids on the WJ-MSCs, the karyotype was assessed in treated cells vs. untreated control. WJ-MSCs were seeded in 6-well plates (15 × 10^4^ cells/well) and treated with GO or GO-amino acids (100 μg/ml) for 24 h (n = 3). Then, 0.1 μg/ml colcemid was added to the WJ-MSCs media and incubated for 3 h. The trypsinized cells were centrifuged at 1500 rpm for 10 min. Next, the cells were treated in a hypotonic solution (0.56% KCl) for 20 min at 37 °C and fixed in a freshly prepared 3:1 mixture of methanol and acetic acid. After seeding the WJ-MSCs onto glass slides, the slides were dried and soaked in diluted 1.5% trypsin in PBS solution. The slides were washed with PBS three times and soaked in Giemsa staining solution for 4 min. Finally, GenAysis software was used to analyze chromosomal structural aberrations [29].

### 3.6 Statistics

The obtained results were recorded as mean ± standard deviation (SD) The comet scoring was calculated using image J website (https://imagej.nih.gov/ij/). One-way ANOVA with Dunnett's multiple comparison tests was employed to investigate the statistical significance of the results using GraphPad software ver. 6.0. Statistical significance was determined at α = 0.05 (P < 0.05).

## 4 Result and discussion

### 4.1 Characterization of GO-amino acids

#### 4.1.1 FT-IR analysis

**Fig. 1:**
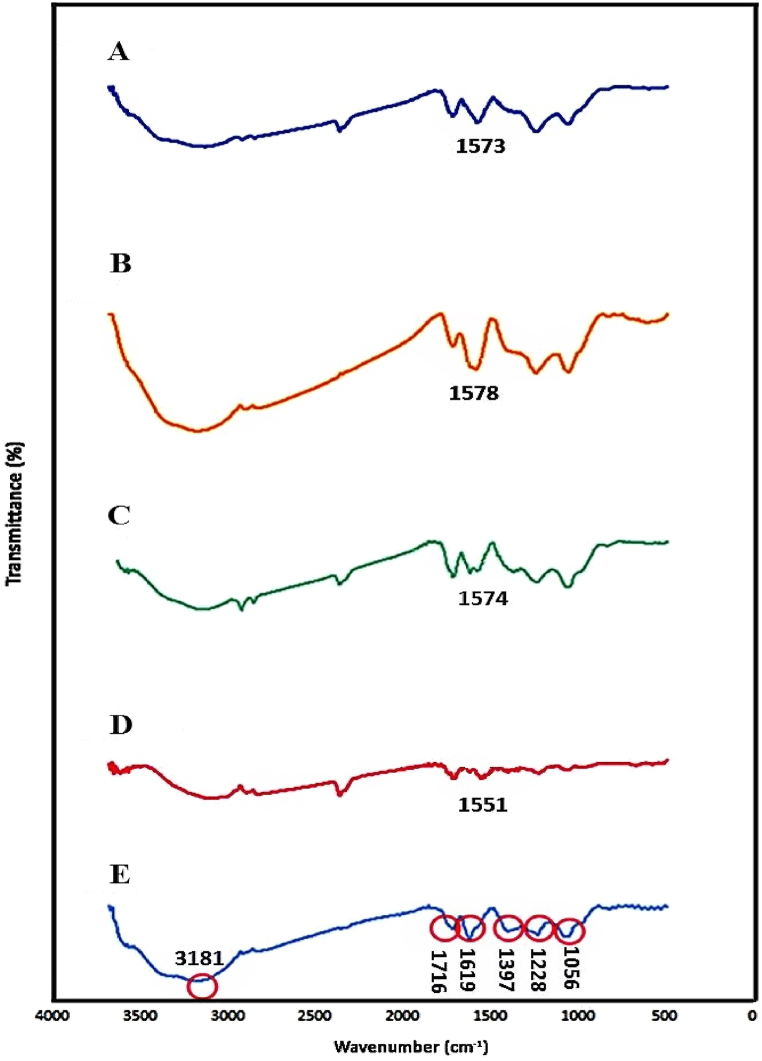
FT-IR spectra of tyrosine-functionalized GO (GO-Tyr, A), methionine-functionalized GO (GO-Met, B), aspartic acid-functionalized GO (GO-Asp, C), lysine-functionalized GO (GO-Lys, D), and unmodified GO (GO, E). *Red circles are characteristic absorption bands for unmodified GO.

The FT-IR spectra of various GO-amino acids were compared to unmodified GO (Fig. 1). Unmodified GO revealed several distinct bands at 3100–3400, 1716, 1619, and 1397 cm^−1^, attributed to the –OH stretching vibration of hydroxyl and carboxylic acid moieties, C=O stretching bands of carboxylic acids, asymmetrical stretching bands of COO, and in-plane bending bands of C–OH, respectively. Furthermore, the bands identified at 1228 and 1056 cm^−1^ are attributed to C–O stretching of epoxy and alkoxy groups, respectively [30,31]. The presence of these oxygen-containing functional groups on the GO surface confirms graphene oxidation [21,32]. Regarding the FTIR spectra obtained from the various GO-amino acids, all distinct bands of GO showed a slight shift in their wavenumber to lower frequencies after the introduction of various amino acids onto the GO nanosheets, which is consistent with the findings of other studies [31,33]. The C=O stretching bands at 1573, 1578, 1574, and 1551 cm^−1^ (Fig. 1) are attributed to amide and carboxylate stretching vibrations of GO-Lys, GO-Asp, GO-Met, and GO-Tyr, respectively, confirming the successful covalent attachment of GO nanosheets by amino acids.

#### 4.1.2 Raman spectroscopy

**Fig. 2:**
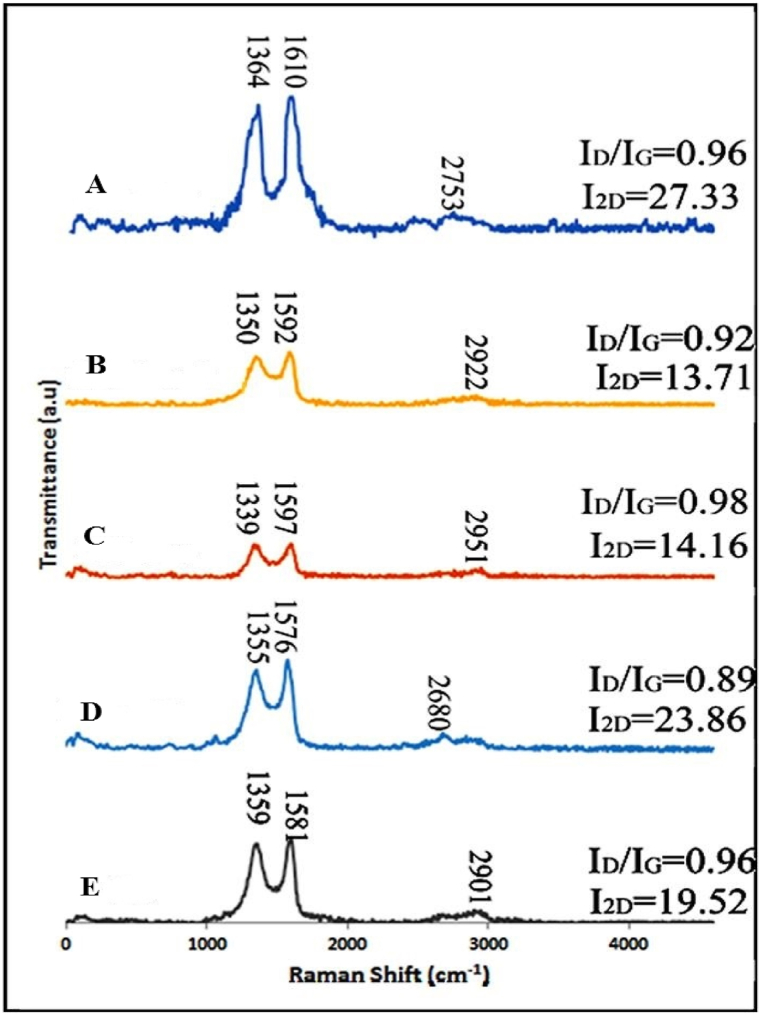
Raman spectra of tyrosine-functionalized GO (GO-Tyr, A), methionine-functionalized GO (GO-Met, B), lysine-functionalized GO (GO-Lys, C), aspartic acid-functionalized GO (GO-Asp, D), and unmodified GO (GO, E).

The structural changes in the GO nanosheets after functionalization with various amino acids were studied using Raman spectroscopy (Fig. 2). Two main and well-known bands of the GO Raman spectrum, namely the D- and G-bands, were observed at 1359 and 1581 cm^−1^, respectively. Generally, the intensity ratio of D- and G-bands (I_D_/I_G_) is employed to ascertain the amount of sp^2^ carbon as well as the degree of covalent functionalization as similarly reported for graphene-based materials in the literature [34,35]. As shown in Fig. 2, the I_D_/I_G_ ratio of GO, GO-Asp, GO-Lys, GO-Met, and GO-Tyr were 0.96, 0.89, 0.98, 0.92, and 0.96, respectively. According to our previous study, the I_D_/I_G_ ratio for pure graphite is found to be 0.1 [21], indicating that sp^2^ carbon is more dominant. The high I_D_/I_G_ values for GO and various GO-amino acids in this study when compared to graphite are due to defects caused by forming sp^3^-bonded carbon during oxidation and functionalization [12]. Generally, these findings indicated that GO was multilayered and functionalization with various amino acids occurs primarily on previously existing defect sites on the GO interface; thus, there was no substantial effect on the concentration of sp^2^ and sp^3^ bonds, and the degree of disorder was similar to GO, indicating no substantial structural change [36].

#### 4.1.3 Elemental analysis

The elemental **(**CHNS) analysis was used to investigate the weight percent of various elements in the samples (GO, GO-Asp, GO-Met, GO-Tyr, and GO-Lys). Table 1 exhibits the elemental weight percentages of carbon (C), nitrogen (N), oxygen (O), and sulfur (S) before and after GO functionalization, confirming the GO modification. For example, the detection of the sulfur atoms in GO-Met (up to 6%) can be attributed to the successful attachment of methionine to GO. Furthermore, the nitrogen contents of GO-Asp, GO-Met, GO-Tyr, and GO-Lys were found to be 2.26%, 4.03%, 2.08%, and 2.87%, respectively, generally confirming the GO modification with the studied amino acids, while no nitrogen was detected in the unmodified GO nanosheets.

**Table 1:** Weight percentages of various elements as determined by elemental (CHNS) analysis. Aspartic acid-functionalized GO (GO-Asp), lysine-functionalized GO (GO-Lys), methionine-functionalized GO (GO-Met), and tyrosine-functionalized GO (GO-Tyr) vs. unmodified GO.

Sample	C%	N%	O%	S%
**GO**	63.94	0.29	35.38	0.4
**GO-Asp**	70.64	2.26	26.76	0.34
**GO-Met**	67.67	4.03	21.83	6.47
**GO-Tyr**	68.20	2.08	29.22	0.49
**GO-Lys**	65.48	2.87	31.21	0.44

#### 4.1.4 Morphology

**Fig. 3:**
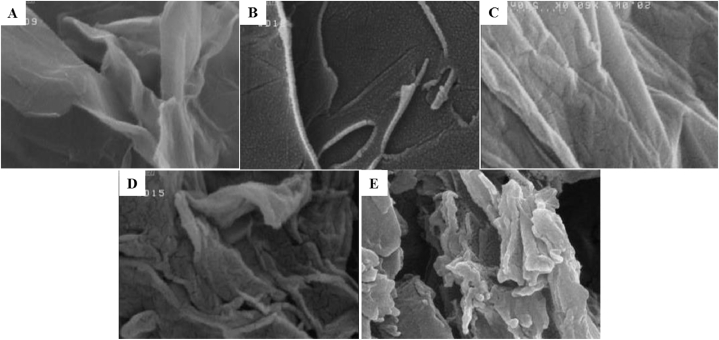
FE-SEM micrographs of unmodified GO and different amino acid functionalized graphene. Unmodified GO (GO, A), methionine-functionalized GO (GO-Met, B), aspartic acid-functionalized GO (GO-Asp, C), lysine-functionalized GO (GO-Lys, D), and tyrosine-functionalized GO (GO-Tyr, E).

The surface morphology of unmodified GO and GO-amino acids was investigated by FE-SEM microscopy. According to Fig. 3, GO revealed a flake-like structure with a smooth surface and large thickness [12]. The surface of GO-amino acids, on the other hand, was rougher and more folded, with tiny particles on the surface, confirming the GO modification by amino acids. Furthermore, the transparency of graphene layers in GO-amino acids (especially GO-Lys and GO-Tyr) was much lower than unmodified GO, which can be attributed to the amino acid attachment on the GO nanosheets, which is consistent with the literature [21,37].

#### 4.1.5 Zeta potential and hydrodynamic diameter

As shown in Table 2, the mean hydrodynamic sizes of GO-amino acids were in the range of 260–480 nm. They were lower in GO-Asp and GO-Tyr, but higher in GO-Lys and GO-Met. Importantly, the size distribution (PDI) of GO-amino acids significantly decreased when compared to GO nanosheets. This could be attributed to attached amino acids which make GO more water dispersible. In other words, the presence of these functional groups in GO-amino acids facilitates GO dispersion in aqueous media due to the hydrophilic nature of surface functionalities and interactions with biological targets [38,39]. Zeta potential was utilized to assess the colloidal stability of various GO-amino acid dispersions. The zeta potential of GO and GO-amino acids were investigated in DI water, phosphate buffer (pH = 7), and DMEM-F12 culture medium. The presence of negatively charged carboxylic acid groups on the surface of GO causes negative zeta potentials [21]. Table 3 displays that the negative zeta potential of GO increased in GO-Asp, GO-Met, and GO-Tyr samples and the charge reversed to a positive zeta potential in GO-Lys in distilled water. GO samples were similarly more negatively charged in phosphate buffer (pH 7) than other media, resulting in high colloidal stability in this medium. The presence of a more negatively charged surface on GO-Asp may be due to the incorporation of two aspartic acid carboxylate groups onto the GO surface. In the DMEM F12 medium, the zeta-potential was similarly lowered to −15 to −19 mV possibly due to the surface adsorption of culture medium components. Overall, the absolute value of the zeta potential increased after GO functionalization with various amino acids, implying that the GO-amino acid dispersion stability was significantly improved [12].

**Table 2:** Hydrodynamic diameter of GO functionalized with different amino acids (n = 3).

Sample	Mean size ±SD	PDI
**GO**	315.3 ± 37.4	0.33
**GO-Asp**	257.9 ± 35.1	0.26
**GO-Met**	380.1 ± 58.0	0.13
**GO-Tyr**	266.4 ± 22.1	0.10
**GO-Lys**	482.0 ± 15.8	0.13

**Table 3:** Zeta potentials of GO and GO functionalized with various amino acids in water, phosphate buffer (pH = 7.4), and DMEM F12 culture medium.

Sample	GO	GO-Lys	GO-Tyr	GO-Asp	GO-Met
**DI Water**	−9.4 ± 4.1	+8.9 ± 3.2	−25.4 ± 5.3	−53.9 ± 0.3	−51.4 ± 1.1
**Phosphate buffer (pH = 7.4)**	−78.6 ± 1.1	−68.0 ± 0.8	−81.8 ± 0.9	−83.5 ± 1.1	−77.7 ± 0.6
**DMEM F12 culture medium**	−18.9 ± 0.4	−17.6 ± 1.4	−15.8 ± 4.8	−16.7 ± 1.5	−15.1 ± 0.3

### 4.2 WJ-MSCs characterization by flow cytometry

**Fig. 4:**
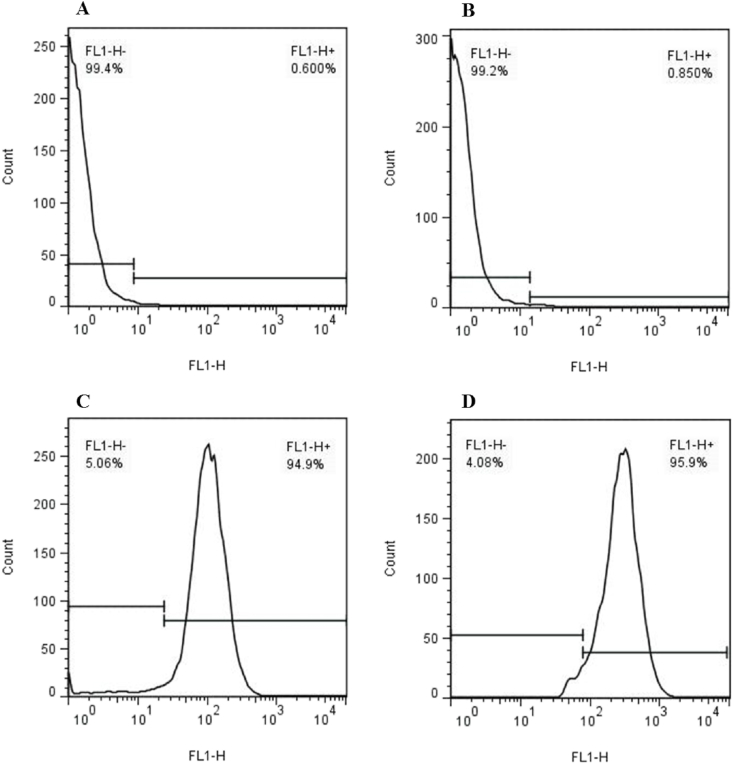
Flow cytometry results showed that the recovered cells were negative for CD34 (0.6%, A), CD45 (0.85%, B) and positive for CD44 (94.9%, C) and CD90 (95.9%, D).

Flow cytometry revealed that WJ-MSCs expressed stromal markers (CD90 and CD44) but not hematopoietic markers (CD34 and CD45) (Fig. 4). The findings are consistent with the literature [40], which shows that WJ-MSCs express CD105, CD44, CD73, and CD90 but not hematological cell surface markers such as CD34, CD19, CD45, CD11a, and HLA DR (human leukocyte antigen).

### 4.3 Cell morphology and viability assays

**Fig. 5:**
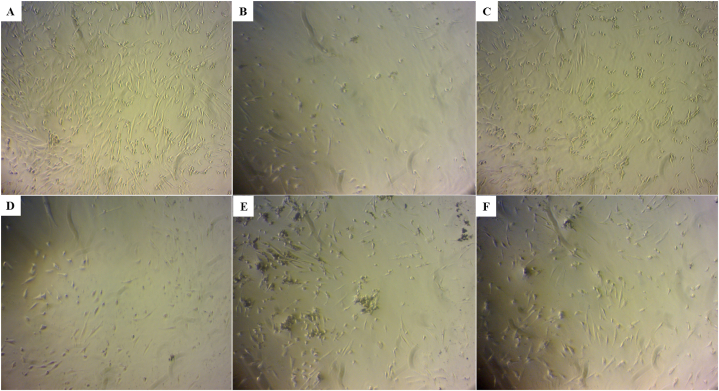
The morphology of Wharton's jelly-mesenchymal stem cells (WJ-MSCs): Control (A), untreated and treated with GO (B), aspartic acid-functionalized GO (GO-Asp, C), lysine-functionalized GO (GO-Lys, D), tyrosine-functionalized GO (GO-Tyr, E), and methionine-functionalized GO (GO-Met, F) at the concentration of 100 μg/ml for 48 h.

GO can cause dose-dependent oxidative stress in cells and induce cellular toxicity as reported by Chang et al. [41]. Oxidative stress is a well-recognized toxicological mechanism of various nanoparticles like GO [42,43]. Amino acid modifications are performed to improve biocompatibility, cellular uptake, and selective activity, as well as to modulate surface functionality and colloidal stability of various nanomaterials [[44], [45], [46], [47]]. In the present study, the effect of GO and GO-amino acids on cell morphology and viability was examined by light microscope, Trypan blue exclusion, and Alamar blue assays. As shown in Fig. 5, cell density was substantially decreased by GO and GO-Lys, and to a lesser extent by GO-Tyr and GO-Met. Furthermore, GO and GO-Lys treated cells had a more circular shape than untreated cells with elongated cell morphology. Importantly, when compared to GO, GO-Asp treated cells significantly increased WJ-MSC spread and maintained elongated cell morphology.

**Fig. 6:**
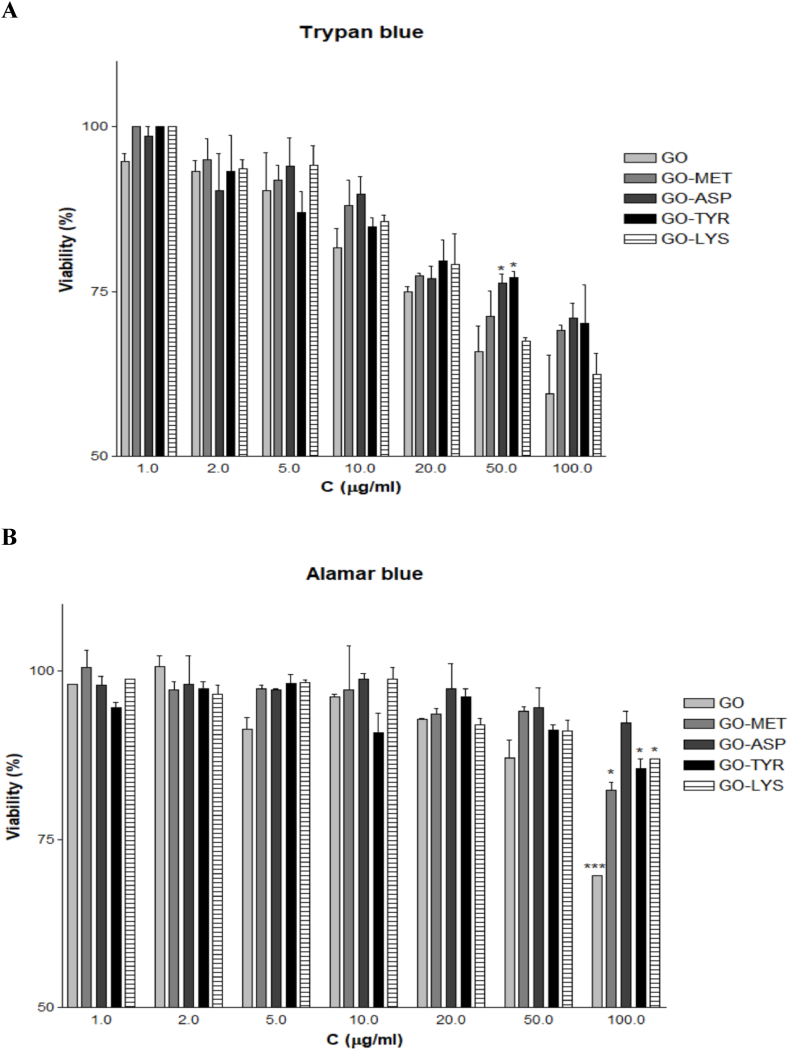
WJ-MSC viability in various concentrations of GO and GO-amino acids either with Trypan blue (A) or Alamar blue (B) cytotoxicity assays after a 48 h incubation. Aspartic acid-functionalized GO (GO-Asp), lysine-functionalized GO (GO-Lys), methionine-functionalized GO (GO-Met), and tyrosine-functionalized GO (GO-Tyr).

GO toxicity has been shown in different cell lines, including human fibroblast (HDF), human gastric cancer (MGC803), breast cancer (MCF-7 and MDA-MB-435), liver cancer (HepG2), and human alveolar basal epithelial cell (A549), all with significant toxicity observed at a concentration of ∼50 μg/ml [41,48,49]. Chowdhury et al. synthesized the oxidized graphene nanoribbons (*O*-GNR) via longitudinal unzipping of carbon nanotubes and evaluated their cytocompatibility in four cell lines; Henrietta Lacks cells derived from cervical cancer tissue (HeLa), National Institute of Health 3T3 mouse fibroblast (NIH-3T3), Sloan Kettering breast cancer (SKBR3) and Michigan cancer foundation-7 breast cancer cell (MCF7). MCF7 and SKBR3 cells exhibited less cytotoxicity than HeLa cells, resulting in substantial cell death even at a low concentration of 10 μg/ml [50]. Therefore, GO cytotoxicity might vary depending on both material properties and cell type. In the present study, Trypan blue exclusion and Alamar blue tests were used to assess GO cytotoxicity in WJ-MSCs. The amino acid modifications resulted in a considerable decrease in the cytotoxicity of GO in various concentrations. Fig. 6A (Trypan blue assay) shows that GO-Asp and GO-Tyr caused the lowest toxicity at the concentration of 50 μg/ml (*P* < 0.05). Similarly, the cytotoxicity of the GO and GO-amino acids was determined by Alamar blue assay (Fig. 6B). Unlike GO showed the highest toxicity, GO-Asp caused the lowest toxicity particularly at 100 μg/ml (*P* < 0.001). Similarly, Das et al. synthesized reduced GO (RGO), which had about 15% less toxicity than unmodified GO at a concentration of 10 μg/ml because of the reduction of oxygen functional groups in RGO [49]. Therefore, amino acid modification can be used to reduce GO cytotoxicity.

### 4.4 Apoptosis and necrosis assay

**Fig. 7:**
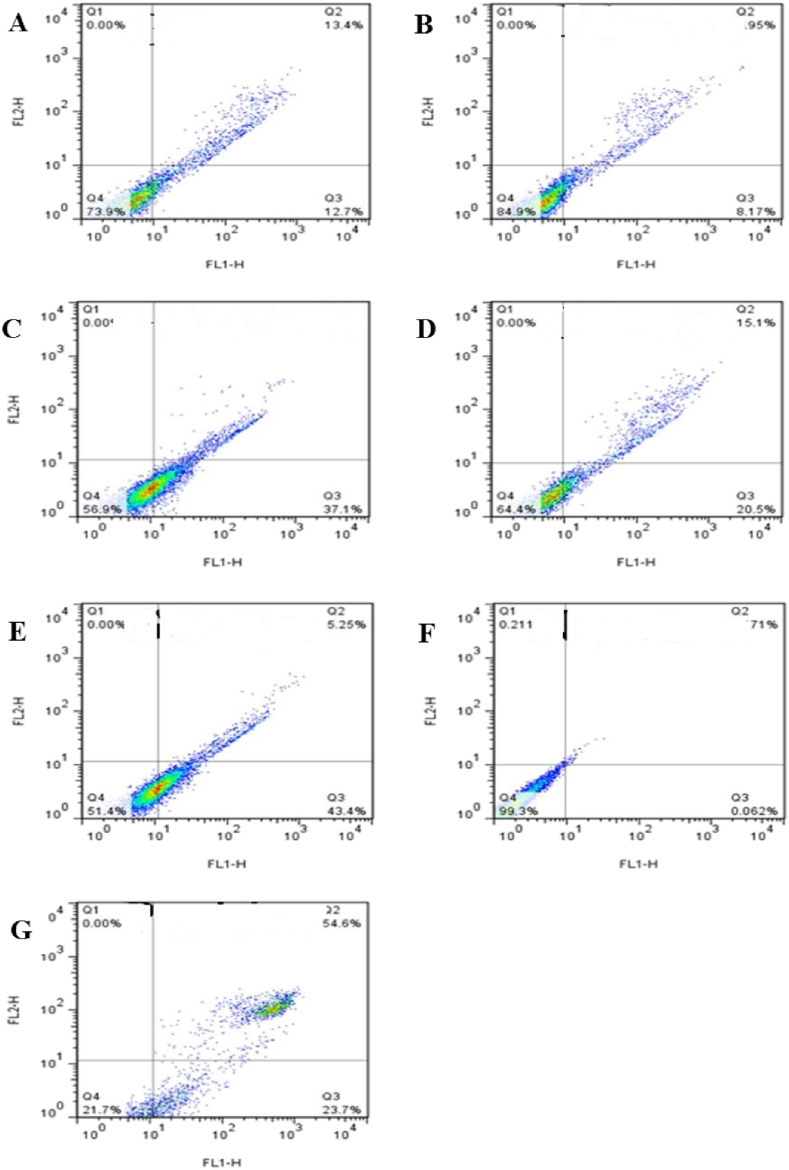
Flow cytogram showing apoptosis assay based on Annexin V–FITC and PI staining of cells after 24 h incubation with GO and different amino acid functionalized GO. Untreated and treated with GO (A), aspartic acid-functionalized GO (GO-Asp, B), methionine-functionalized GO (GO-Met, C), lysine-functionalized GO (GO-Lys, D), tyrosine-functionalized GO (GO-Tyr, E), and Neg. contro (F): untreated negative control; Pos. Control (G): positive control cells incubated in 55 °C for 20 min. Percentages of cells in Q4 region denotes live cells, Q3: early apoptotic, Q2: necrotic cells, Q1: late apoptotic.

**Fig. 8:**
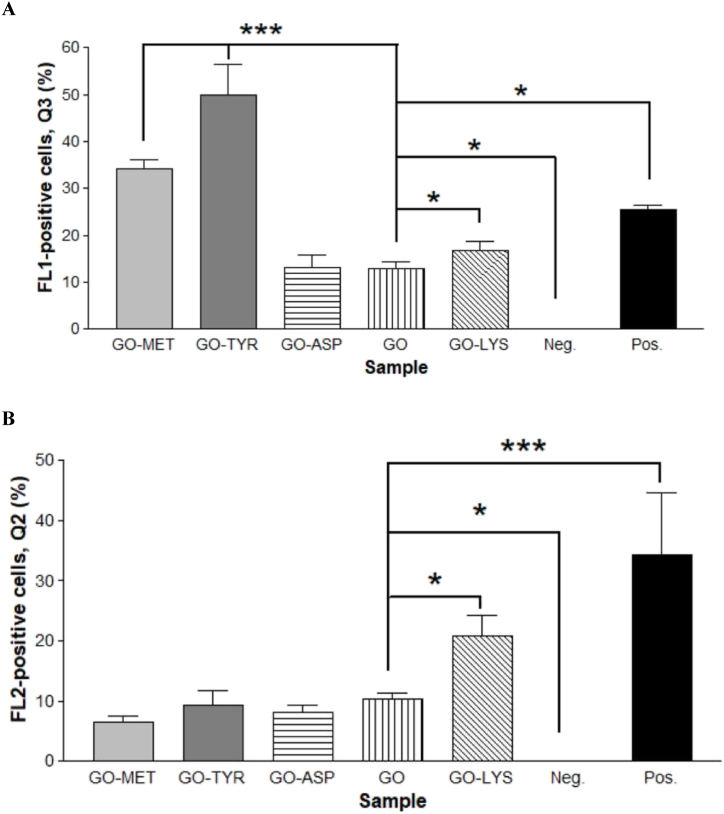
Percentage of apoptotic (Q3 area, A) and dead mesenchymal stem cells (or secondary necrosis - Q2 region, B) after 24 h incubation with GO modified with different amino acids vs. unmodified GO. Aspartic acid-functionalized GO (GO-Asp), lysine-functionalized GO (GO-Lys), methionine-functionalized GO (GO-Met), and tyrosine-functionalized GO (GO-Tyr). Neg. control: untreated negative control; Pos. Control: positive control cells incubated in 55 °C for 20 min * and *** denote P values < 0.05 and <0.001 for differences between GO and other groups, respectively.

To elucidate the mechanism of WJ-MSC cytotoxicity induced by GO and GO-amino acids, the apoptotic (Q3) and necrotic (Q2) deaths were examined by flow cytometric analysis using Annexin V-FITC/PI staining [51,52]. Flow cytograms for cells treated with GO and GO-amino acids revealed different levels of apoptosis and necrosis when compared with untreated negative and H_2_O_2_-treated positive controls (Fig. 7). As shown in Fig. 8A, GO caused the lowest apoptosis (12.8%); however, GO-Tyr and GO-Met caused the highest apoptosis rate (49.8% and 34.1%, respectively) (P < 0.001). In addition, the apoptosis increased in GO-Lys compared to the negative control (P < 0.05). Because of the positive charge of lysine and its affinity to bind to the cell membrane, GO-Lys had higher necrosis (P < 0.05) than the GO group. When WJ-MSCs were incubated with GO-Asp, GO-Tyr, or GO-Met, the induced necrosis was not statistically significant when compared to the untreated control group (Fig. 8B). Consistent with the present results, Sasidharan et al. reported that pristine graphene accumulated on the cell membrane causing high oxidative stress leading to apoptosis, while carboxyl-functionalized hydrophilic graphene was internalized by cells without causing toxicity and apoptosis induction [53]. Overall, total death cells were significantly lower in the GO-Asp group, indicating the highest cytocompatibility.

### 4.5 Alkaline comet assay

**Fig. 9:**
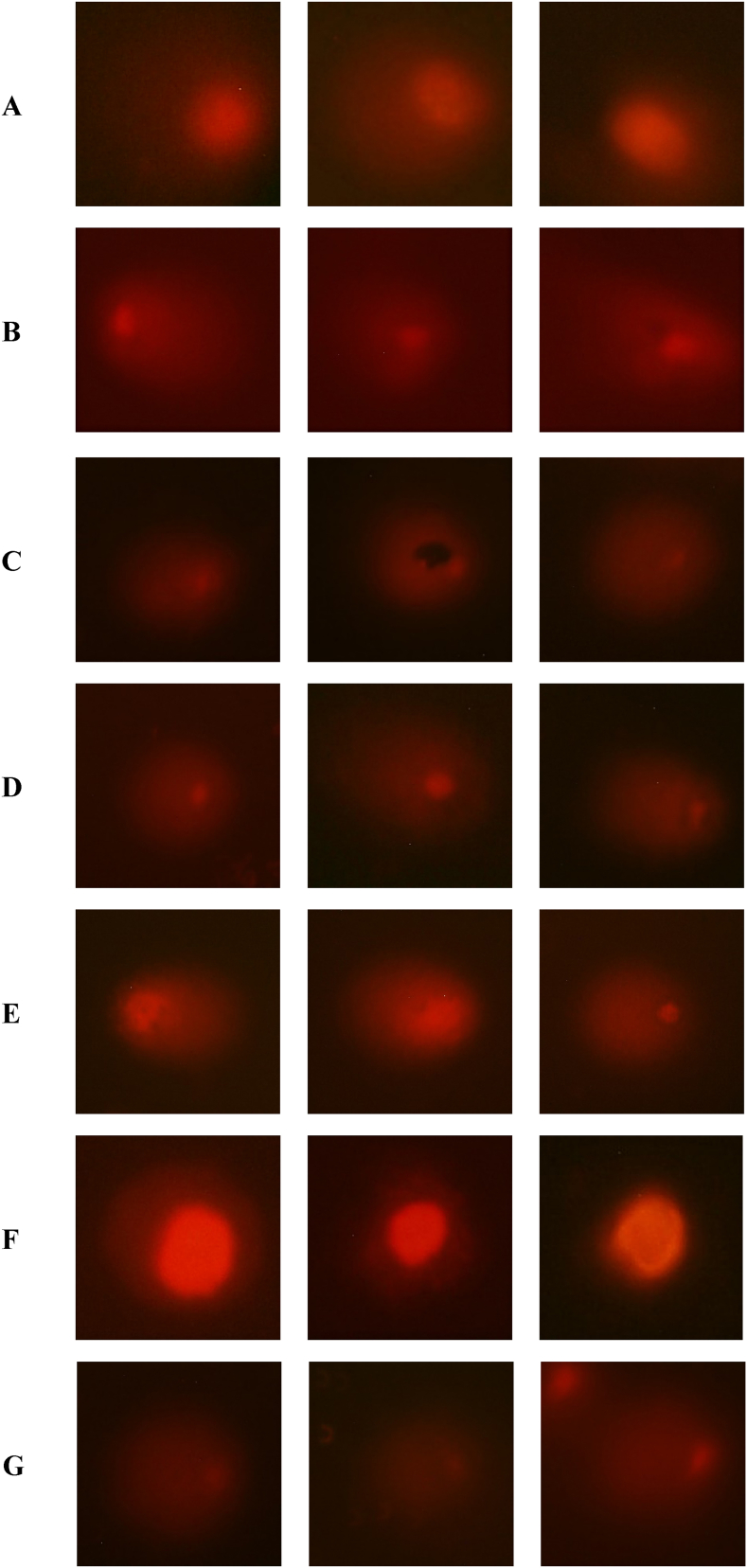
Comparison of DNA damage images as detected by the comet assay on WJ-MSCs after exposure to aspartic acid-functionalized GO (GO-Asp, A), unmodified GO (B)), lysine-functionalized GO (GO-Lys, C), methionine-functionalized GO (GO-Met, D) tyrosine-functionalized GO (GO-Tyr, E), neg. control (F): untreated negative control; pos. Control: positive control exposed to 200 μM H_2_O_2_ (G). Images obtained after the comet assay process. Magnification 400 × .

**Fig. 10:**
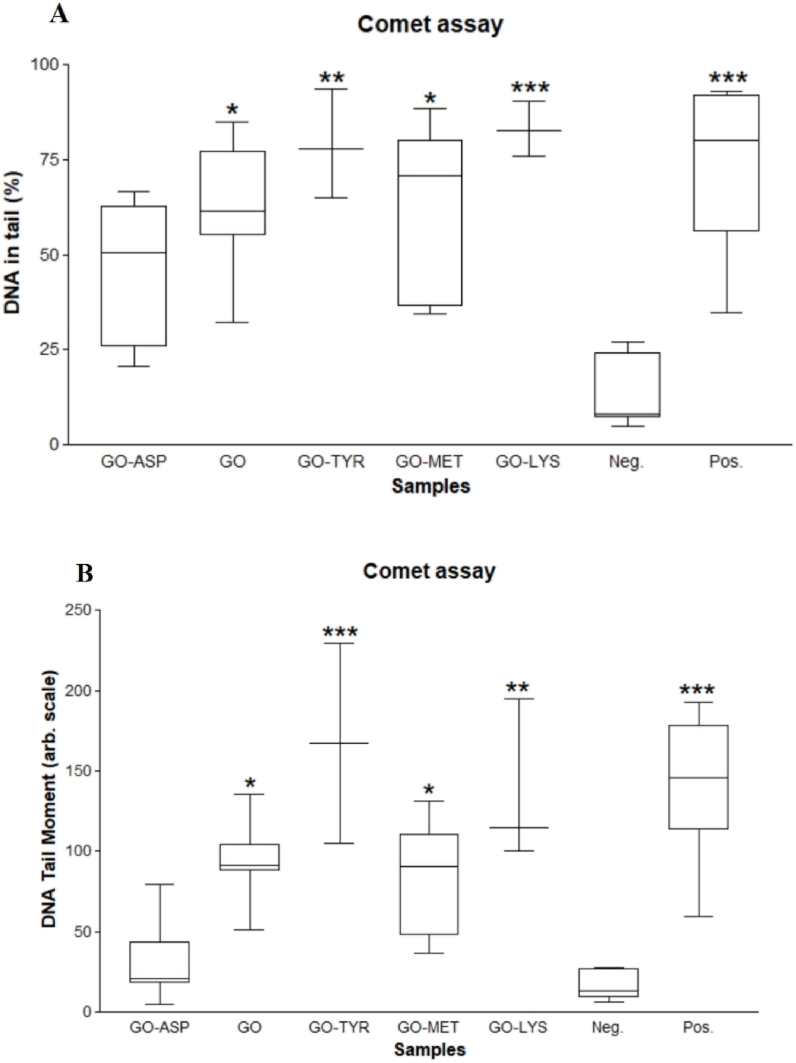
A boxplot of (A): % DNA in tail and, (B): Tail moment of groups exposed to GO, aspartic acid-functionalized GO (GO-Asp), lysine-functionalized GO (GO-Lys), methionine-functionalized GO (GO-Met), and tyrosine-functionalized GO (GO-Tyr). Neg: negative untreated control group, Pos: positive control group exposed to 200 μM H_2_O_2_. Each box represents an interquartile range and a median line. Whiskers also display the minimum and maximum values. *, **, and *** denote P values < 0.05, <0.01, and <0.001 for differences between the negative control and other groups, respectively.

DNA is prone to oxidation, which causes single and double-strand breaks, DNA-protein cross-links, and inter/intra-strand cross-links [54,55]. The overproduction of free radicals can cause oxidative damage to lipids and proteins, which can lead to DNA damage [56,57]. To investigate whether GO and GO-amino acids could be used in the field of cell therapy, their genotoxicity was tested. The Comet assay was used to examine the damage caused by GO and GO-amino acids on DNA [58,59]. Fluorescence microscope images (Fig. 9) show varying degrees of stained DNA breaks in the form of a comet after single cell electrophoresis. The percentage of DNA in the tail showed a significant increase in GO-Lys (P < 0.001) and GO-Tyr (P < 0.01). Unmodified GO and GO-Met showed increased DNA in the tail (P < 0.05); however, GO-Asp did not, indicating high DNA compatibility (Fig. 10A). The tail moment (% DNA in the tail × tail length) was significantly higher in GO-Lys (P < 0.01) and GO-Tyr (P < 0.001), suggesting a genotoxic effect on WJ-MSCs (Fig. 10B). GO and GO-Met also showed a significant increase in the tail moment (P < 0.05). Similarly, Akhavan et al. revealed that GO has concentration-dependent genotoxicity, such that at concentrations less than 200 μg/ml, DNA cleavage and chromosome damage were not significantly different from the negative control, but significantly increased at 2000 μg/ml concentration [60]. The lysine and tyrosine modified GOs caused the most DNA damage. Cationic amino acids with positive charges, such as lysine, can bind to negatively charged biological materials such as DNA, cell membrane phospholipids, and cell membrane proteins [[61], [62], [63]]. *In vitro* and *in vitro* studies have also shown that tyrosine can cause oxidative stress and DNA damage [56,64]. According to the comet assay results, modifying GO with methionine did not result in a significant change in tail length or the percentage of DNA in the tail. Due to its function in the metabolism of carbon and DNA methylation, methionine has an impact on DNA integrity [65]. On the other hand, excessive amounts of methionine are toxic, can harm a variety of tissues and organs, and cause oxidative stress [66,67]. GO-Asp did not differ significantly from the negative control group, but when compared to GO, the tail length and percentage of DNA in the tail were significantly reduced in GO-Asp, indicating non-significant genotoxicity of GO-Asp. Therefore, depending on the amino acid used, amino acid modification can change GO genotoxicity.

### 4.6 Karyotyping assay

**Fig. 11:**
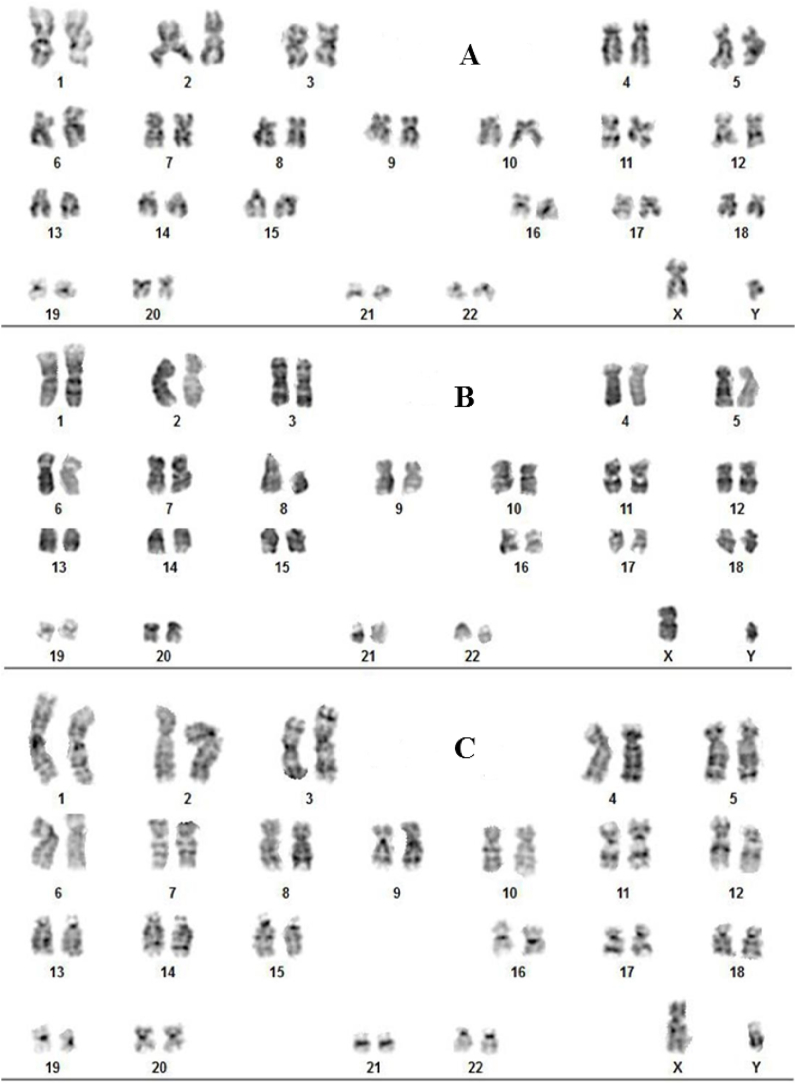

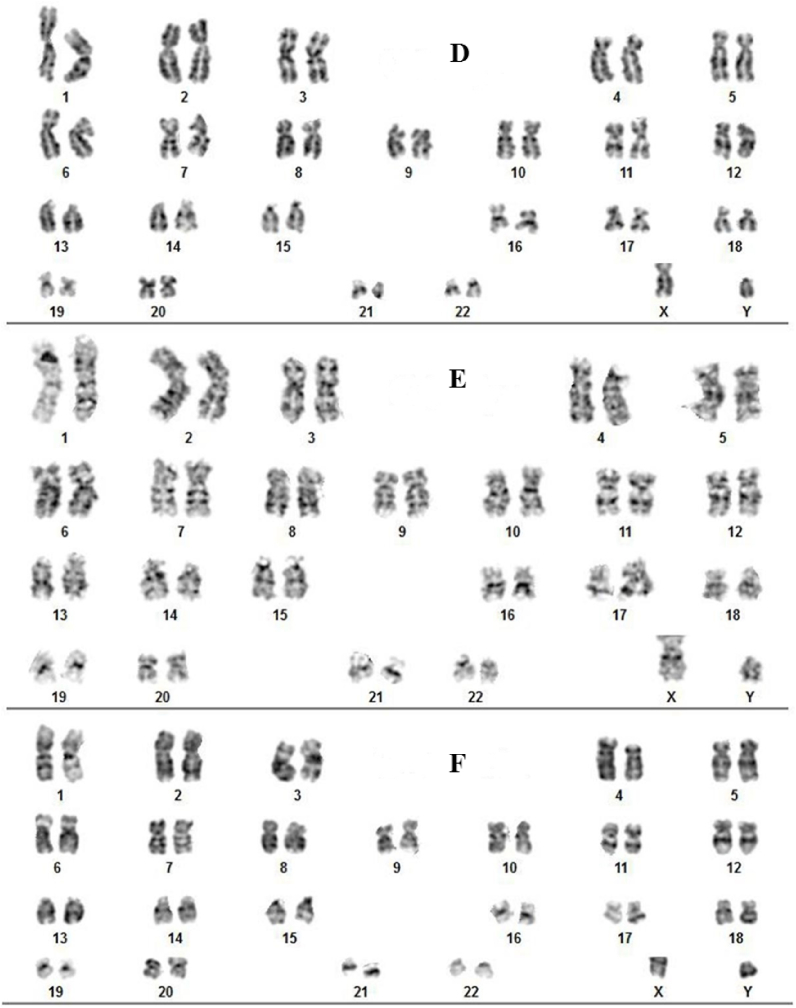
The karyotyping results of WJ-MSCs treated with Neg: negative untreated control group (A), unmodified GO (B) and GO-amino acids. Aspartic acid-functionalized GO (GO-Asp, C), lysine-functionalized GO (GO-Lys, D), tyrosine-functionalized GO (GO-Tyr, E), and methionine-functionalized GO (GO-Met, F).

The karyotyping assay was carried out to examine whether functionalized GO (GO-Asp, GO-Met, GO-Tyr, and GO-Lys) brought about toxic effects on WJ-MSC chromosomes [68,69] in comparison with unmodified GO. As shown in Fig. 11, the results of classical G-banding showed normal diploid karyotype in each group at the corresponding concentration of 100 μg/ml. Cherian et al. synthesized *Halomonas Maura-*reduced GO (MRGO) that caused chromatid breaks and chromosome gap at a high concentration (600 μg/ml), but at a concentration of 100 μg/ml, no significant difference was observed with the untreated control [70]. Akhavan et al. investigated *in vivo* dose-dependent effects of nanoscale graphene oxide (NGO) sheets in Balb/C mice. For the injected concentrations ≤200 μg/ml, no remarkable DNA fragmentation was found. However, considerable chromosomal aberrations were observed at the high concentration of 2000 μg/ml [60]. In another study, they reported that GO nano-ribbons can cause DNA fragmentation as well as chromosomal aberrations even at low concentrations and after a short exposure time [11]. Also, Hanan et al. found that oral administration of GO nanoparticles for one or five consecutive days at the three dose levels 10, 20, or 40 mg/kg significantly increased the micronuclei and DNA damage levels in a dose-dependent manner in mice bone marrow cells [71]. Considering the exposure time and GO concentration, the current study finding is consistent with earlier reports.

## 5 Conclusion

In this study, we evaluated the cytotoxicity and genotoxicity of GO synthesized by the Hummers' method after modifying with amino acids including Lys, Met, Tyr, and Asp. The FT-IR and EDX results confirmed the successful covalent attachment of GO nanosheets by amino acids and RAMAN findings indicated that GO was multilayered and functionalized with various amino acids. In addition, the mean hydrodynamic sizes of GO-amino acids were in the range of 260–480 nm and their surface became rougher and more folded than GO. The GO-amino acid dispersion stability was superior to GO based on increased zeta potentials after the amino acid functionalization. Trypan Blue and Alamar Blue cytotoxicity methods on MSCs showed that the dose-dependent cytotoxicity of GO significantly decreased for GO modified with different amino acids. Annexin V staining method which evaluated the apoptotic and necrotic death showed that GO treated with Tyr and Met revealed the highest rate of apoptosis, and Lys-modified GO exhibited remarkable necrosis. In contrast, GO-Asp showed the lowest cytotoxicity and apoptosis induction. COMET and karyotyping techniques evaluated the extent of DNA and chromosomal damage in MSCs. In the karyotyping method, no chromosomal damage was reported, but in the comet assay, the samples modified with Tyr and Lys showed the most DNA damage. Finally, the Asp-modified GO caused the least levels of cellular and genetic damage to MSCs and can be a good choice in biological applications without a compromise in carboxylic acid functionality.

## Author contribution statement

Ali Mohammad Tamaddon: Conceived and designed the experiments; Analyzed and interpreted the data; Contributed to materials, analysis tools, or data. Sedigheh Borandeh: Conceived and designed the experiments. Samira Sadat Abolmaali, Negar Azarpira: Conceived and designed the experiments; Contributed to materials, analysis tools, or data. Rahman Bashiri: Analyzed and interpreted the data; Performed the experiments. Haniyeh Najafi, Khadijeh Mousavi: Analyzed and interpreted the data; Performed the experiments; Wrote the paper. Mahdokht H Aghdaie: Analyzed and interpreted the data. Mahboobeh Jafari, Mina Shafiee: Contributed to materials, analysis tools, or data; Wrote the papper.

## Data availability statement

Data will be made available on request.

## Declaration of competing interest

The authors declare that they have no known competing financial interests or personal relationships that could have appeared to influence the work reported in this paper.
